# The Clinical Efficacy and Cardiotoxicity of Fixed-Dose Monthly Trastuzumab in HER2-Positive Breast Cancer: A Single Institutional Analysis

**DOI:** 10.1371/journal.pone.0151112

**Published:** 2016-03-08

**Authors:** Yi-Ying Wu, Tzu-Chuan Huang, Tsung-Neng Tsai, Jia-Hong Chen, Ming-Shen Dai, Ping-Ying Chang, Ching-Liang Ho, Ren-Hua Ye, Tsai-Rong Chung, Yeu-Chin Chen, Tsu-Yi Chao

**Affiliations:** 1 Division of Hematology/Oncology, Department of Medicine, Tri-Service General Hospital, Taipei City, Taiwan, Republic of China; 2 Graduate Institute of Life Sciences, National Defense Medical Center, Taipei City, Taiwan, Republic of China; 3 Division of Cardiology, Department of Medicine, Tri-Service General Hospital, Taipei City, Taiwan, Republic of China; 4 Graduate Institute of Clinical Medicine, College of Medicine, Taipei Medical University, Taipei City, Taiwan, Republic of China; 5 National Institute of Cancer Research, Miaoli County, Taiwan, Republic of China; 6 Division of Hematology and Oncology, Department of Medicine, Taipei Medical University, Shuang-Ho Hospital, New Taipei City, Taiwan, Republic of China; University of North Carolina School of Medicine, UNITED STATES

## Abstract

**Objective:**

Trastuzumab-containing treatment regimens have been shown to improve survival outcomes in HER2-positive breast cancer (BC). It is much easier to infuse a fixed one-vial dose to every patient on a regular schedule in the general clinical setting. The aims of this study were evaluating the efficacy of a 440 mg fixed-dose of trastuzumab administered on a monthly infusion schedule, and the risk factors for cardiac events.

**Patients and methods:**

We retrospectively reviewed data from 300 HER2-positive BC patients in our institute: 208 were early-stage BC patients undergoing adjuvant trastuzumab treatment, and 92 were metastatic BC patients treated with trastuzumab infusions until disease progression. There were 181 patients receiving regular trastuzumab infusions every 3 weeks (Q3W; 8 mg/kg loading dose followed by 6 mg/kg every 3 weeks), and the other 119 patients were treated monthly with a fixed 440 mg dose (QM; fixed 440 mg every 4 weeks).

**Results:**

The medians of progression-free survival (PFS) and overall survival (OS) in the adjuvant setting were not reached in both treatment groups. In the metastatic setting, there was no significant difference between groups in PFS or OS. The median time to significant cardiovascular (CV) dysfunction was 4.54 months. The incidence of congestive heart failure requiring medication in our cohort was 3.4%.

**Conclusion:**

In our study, we found that fixed-dose monthly trastuzumab was feasible and effective. In addition, the CV risk was not higher with the fixed-dose protocol. This treatment modality could lower the cost and was easier to implement in clinical practice. Larger prospective randomized studies with longer-term follow up are needed to confirm our results.

## Introduction

Trastuzumab is a humanized monoclonal antibody that binds to the extracellular domain of the HER2 transmembrane growth factor receptor [1]. Around 20% to 30% of all invasive breast carcinomas are HER2-positive, and most of these have aggressive clinical behavior along with a poor outcome [2]. Several clinical trials have already shown that treating HER2-overexpressing metastatic breast cancer (BC) with trastuzumab, either alone or in combination with chemotherapy, significantly improves time to progression, duration of response, and survival [3, 4]. Trastuzumab has also been shown to be an effective adjuvant therapy for HER2-positive early-stage BC patients [5, 6].

The most common side effect of trastuzumab is cardiomyopathy, reported to affect around 2.8% to 3.3% of patients [7]. Most clinical trials monitor heart function via resting left ventricular ejection fraction (LVEF), and the incidence of trastuzumab discontinuation due to cardiac events is low [8]. Some researchers have found that previous treatment with anthracycline regimens, menopause status, radiotherapy, and the frequency of trastuzumab infusion might be associated with a higher incidence of cardiomyopathy [9]. The cardiotoxicity appears to be reversible, and left ventricular dysfunction often normalizes after withdrawal of trastuzumab [10].

Each clinical trial has used a different dosage and infusion frequency of trastuzumab. The most common dosage was 4 mg/kg for the loading dose followed by 2 mg/kg per week [6]. In the HERceptin Adjuvant (HERA) trial [11], the dosage was adjusted to 8 mg/kg for the loading dose followed by 6 mg/kg every 3 week for the clinical convenience. However, Pearson et al. reported 24% of trastuzumab dispensed was discarded, at a cost of $21.1 million Australian [12]. It is important and required immediate attention to minimize drug wastage. In Taiwan, one vial of Herceptin contains 440 mg. The waste of expensive trastuzumab can be occurred and also becomes a pharmacoeconomic stress when a patient receives a weight-based infusion weekly or triweekly protocol. Therefore, some BC patients receive fixed-dose monthly trastuzumab of 440mg in order to reduce the frequency of drug administration and even to lower their financial stress. However, the efficacy of this real-world practice is not known yet.

In this retrospective study, we try to analyze the outcomes of patients with early-stage and metastatic BC treated with either a fixed-dose (440 mg trastuzumab given as a monthly infusion) or with a weight-based infusion triweekly protocol. We studied the efficacy of the fixed-dose monthly trastuzumab dose in our cohort by measuring the time to progression and overall survival in our study cohort. We also evaluated possible risk factors for cardiotoxicity.

## Materials and Methods

### Study population

We retrospectively collected data from HER2-positive BC patients treated with trastuzumab at Tri-Service General Hospital from September 2006 to December 2014. Those treated with trastuzumab containing dual blockade, enrolled in clinical trials, or unfit for treatment were excluded. The primary objective of this study was to evaluate the efficacy of a 440 mg fixed-dose of trastuzumab on a monthly infusion schedule (QM group) compared to a triweekly weight based dose protocol (Q3W group) in adjuvant and metastatic setting. The secondary aim was to evaluate risk factors for cardiac events in our cohort.

Each patient completed staging workup including chest radiography, abdominal ultrasonography, and a bone scan. Advanced-stage individuals were examined by positron emission tomography and brain magnetic resonance imaging. The patients’ clinical information and laboratory data were collected and analyzed from medical records. Pathological slides were examined. The expression of estrogen receptor (ER) was determined by immunohistochemical (IHC) staining of paraffin-embedded tumor. HER2 overexpression was defined as an IHC score of 3+ (Dako) or amplification by fluorescence *in situ* hybridization. All of these studies were performed in accordance with the guidelines of the Helsinki Declaration and were approved by the Human Subjects Protection Offices (IRB) of Tri-Service General Hospital.

### Treatment and follow-up modalities

Patients were treated as clinically indicated, and therefore trastuzumab was often combined with chemotherapy. In early-stage BC, the Q3W group received adjuvant trastuzumab treatment with a loading dose of 8 mg/kg, followed by 6 mg/kg every 3 weeks for up to 1 year. In the QM group, trastuzumab was administered as a fixed 440 mg monthly dose for 12 months as adjuvant therapy. In metastatic BC, monthly or triweekly trastuzumab was administered until disease progression.

### Cardiac function evaluation

Echocardiography was performed before treatment and every 3–6 months, as clinically applicable. LVEFs were measured and recorded. Trastuzumab-related cardiotoxicity was classified into five grades as defined in the previous literature [6]:

- Grade I: asymptomatic decline in LVEF of >10% from baseline evaluation.
- Grade II: asymptomatic decrease in LVEF of <50% or ≥20% compared to the baseline value.
- Grade III: heart failure responsive to treatment.
- Grade IV: severe or refractory heart failure, or heart failure requiring intensive medical therapy and/or intubation.
- Grade V: death related to cardiac toxicity.

Treatment with a trastuzumab-containing regimen was discontinued or a trastuzumab rechallenge was considered by the clinical oncologist if the patient had cardiotoxicity of more than grade II severity.

### Statistical analyses

All analyses were performed using SPSS version 18.0 software for Windows (SPSS, Inc.). The significance level was 5% for all analyses. All descriptive data are expressed as the median ± standard deviation. Two-tailed *t* tests and chi-square tests were used to compare the baseline characteristics of the Q3W and QM groups. Log-rank tests and Kaplan-Meier plots were used to analyze each groups’ progression free survival (PFS), overall survival (OS), and time to cardiac events. Cox-regression models were used to evaluate multiple variables. The risk factors for cardiac events were analyzed with the chi-square test.

## Results

### Patient characteristics

Two hundred and eight patients with early-stage HER2-positive BC and 92 patients with HER2-positive metastatic BC were treated with a trastuzumab-containing regimen. The demographic differences between the Q3W and QM groups were compared and are shown in Table 1. All patients were women with a median age of 52 years. The patients in the QM group were slightly younger (*p* = 0.021). The lesion side, radiotherapy plan, and ER positivity rate were similar in the two groups. However, in the adjuvant setting, the Q3W group had a higher proportion of patients with stage IIIA to IIIC disease and a higher proportion of patients who received a chemotherapy regimen containing anthracycline plus taxane than the QM group (31.1% versus 18.6% and 59.7% versus 38.7%, respectively). In the adjuvant setting, the total treatment cycles of trastuzumab were 18 for the Q3W group, and 12 for the QM group. The average dosage of trastuzumab for each cycle was 360 mg in the Q3W group. On the other side, the median treatment cycles were the same in both groups (15 cycles) in the metastatic setting. The average dosage of trastuzumab for each cycle was 336 mg in the Q3W group. There were very few patients who had cardiovascular (CV) risk factors. We used age (>55 years-old), hypertension, diabetes, hyperlipidemia, and smoking to define a total CV risk group. We found that the Q3W and QM groups had relatively similar CV risks (*p* = 0.44).

**Table 1:** The clinical demographic differences between both study groups.

Variables		Q3W	QM	*p* value
Patient number		N = 181	N = 119	
Age (years±SD)		53.2 ±10.4	50.3 ±10.6	0.021
Side (%)	Right	89 (49.2)	48 (40.3)	0.243
	Left	88 (48.6)	66 (55.5)	
	Bilateral	4 (2.2)	5 (4.2)	
Stage (%)	Stage I	32 (17.7)	25 (21.0)	0.013
	Stage IIA	37 (20.4)	23 (19.3)	
	Stage IIB	26 (14.4)	9 (7.6)	
	Stage IIIA	19 (10.5)	6 (5.0)	
	Stage IIIB	8 (4.4)	4 (3.4)	
	Stage IIIC	16 (8.8)	3 (2.5)	
	Stage IV	43 (23.8)	49 (41.2)	
Hypertension (%)		32 (17.7)	23 (19.3)	0.735
Diabetes (%)		17 (9.4)	4 (3.4)	0.043
Smoking (%)		11 (6.1)	4 (3.4)	0.282
Hyperlipidemia (%)		6 (3.3)	3 (2.5)	0.681
ER^a^ (%)		96 (53.0)	52 (43.7)	0.171
Radiotherapy (%)		114 (63.0)	67 (56.3)	0.274
Chemotherapy (%)	Other	19 (10.5)	11 (9.2)	<0.0001
	Taxane^b^	41 (22.7)	13 (10.9)	
	Epirubicin	6 (3.3)	10 (8.4)	
	Doxorubicin	7 (3.9)	39 (32.8)	
	Epirubicin + Taxane	40 (22.1)	12 (10.1)	
	Doxorubicin+ Taxane	68 (37.6)	34 (28.6)	

a. ER: estrogen receptor

b. Taxane included docetaxel or paclitaxel

### Time to progression and overall survival

**Fig 1:**
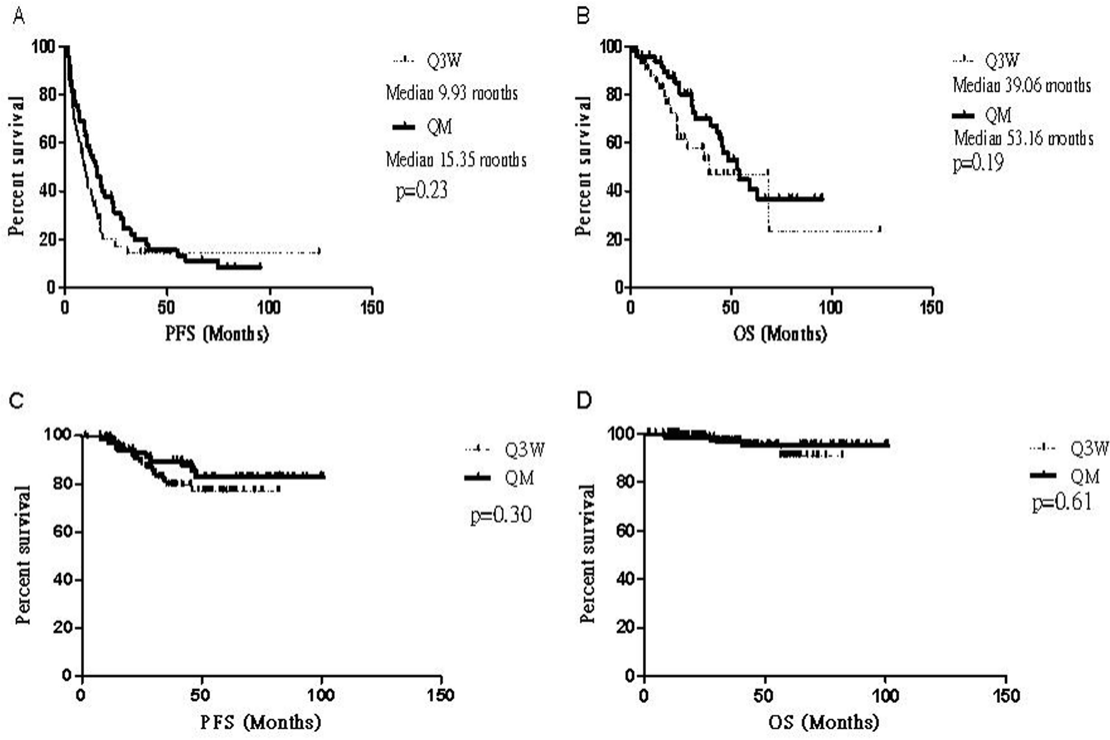
Kaplan-Meier curves comparing the treatment effect in the Q3W and QM groups. (A)There is no significant difference in PFS or OS (B) between the groups in the metastatic cohort. (C)In the adjuvant cohort, the medians of PFS and OS (D) were not reached.

The survival of the metastatic cohort was evaluated with the log-rank test and Kaplan-Meier plots. No significant difference in PFS and OS was noted between the Q3M and QM groups (Fig 1A and 1B; *p* = 0.23 and *p* = 0.19, respectively). We used a Cox-regression model to try to identify possible confounding factors such as age, ER positivity, lymphovascular invasion, and tumor grade. No specific factors were found to influence PFS or OS in the metastatic cohort (data not shown). The medians of both PFS and OS in the adjuvant group were not reached by the end of the study (Fig 1C and 1D; *p* = 0.30 and *p* = 0.61, respectively). However, through analysis with a Cox-regression model, we found that in the adjuvant cohort, the QM group with younger ages had slightly better PFS than the Q3W group (hazard ratio = 2.445, 95% confidence interval = 1.021–5.858, *p* = 0.045).

### CV events

A total of 261 patients were enrolled to analyze the CV risk factors using the Chi-square test for variables such as trastuzumab infusion frequency, previous anthracycline–containing regimens, lesion side, radiotherapy plan, and CV risk factors. Thirty-nine patients were excluded due to irregular echocardiographic evaluation and a lack of medical information about CV risk factors. We found that no factors other than age correlated to cardiac toxicity (Table 2, *p* = 0.032). The incidence of asymptomatic LVEF declined was 10.7%, and there was no significant difference between both groups (*p* = 0.693). Excluding those with asymptomatic patients, the incidence of congestive heart failure (CHF) (>grade II toxicity) in our patients was 3.4%. Six of these patients were in the Q3W group, and three were in the QM group. No cardiac death (grade V toxicity) occurred in our study population.

**Table 2:** The evaluation of trastuzumab-related cadiotoxicity by variable factors.

Variables		Δ EF 10–20% 〔*n*(%)〕	Δ EF >20% or EF <50% 〔*n*(%)〕	CHF need medication 〔*n*(%)〕	CHF need ICU or intubation 〔*n*(%)〕	*P* value
Trastuzumab	Q3W	13(8.2)	2(1.3)	3(1.9)	3(1.9)	0.913
	QM	11(10.7)	2(1.9)	2(1.9)	1(1.0)	
Chemotherapy	Other	2(7.7)	0(0)	1(3.8)	0(0)	0.943
	Taxane*	2(4.4)	1(2.2)	0(0)	0(0)	
	Epirubicin	1(6.7)	0(0)	0(0)	0(0)	
	Doxorubicin	6(14.6)	1(2.4)	1(2.4)	1(2.4)	
	Epirubicin +Taxane	3(7.0)	1(2.3)	1(2.3)	0(0)	
	Doxorubicin + Taxane	10(11.0)	1(1.1)	2(2.2)	3(3.3)	
Stage	Stage I	6(12.0)	2(4.0)	0(0)	0(0)	0.649
	Stage IIA	5(8.6)	0(0)	0(0)	0(0)	
	Stage IIB	4(12.9)	0(0)	1(3.2)	2(6.5)	
	Stage IIIA	3(12.0)	1(4.0)	1(4.0)	1(4.0)	
	Stage IIIB	1(9.1)	0(0)	0(0)	0(0)	
	Stage IIIC	0(0)	1(5.9)	1(5.9)	0(0)	
	Stage IV	5(7.5)	0(0)	2(3.0)	1(1.5)	
Side	Right	12(9.8)	0(0)	1(0.8)	2(1.6)	0.224
	Left	12(9.2)	4(3.1)	3(2.3)	2(1.5)	
	Bilateral	0(0)	0(0)	1(12.5)	0(0)	
Age (years)	<55	18(11.5)	3(1.9)	0(0)	3(1.9)	0.032
	> = 55	6(5.7)	3(1.0)	3(4.8)	1(1.0)	
Hypertension	With	3(6.1)	2(4.1)	2(4.1)	1(2.0)	0.310
	Without	21(9.9)	2(0.9)	3(1.4)	3(1.4)	
Diabetes	With	2(10.0)	0(0)	1(5.0)	0(0)	0.782
	Without	22(9.2)	4(1.7)	4(1.7)	4(1.7)	
Smoke	With	1(7.7)	0(0)	0(0)	0(0)	0.940
	Without	23(9.3)	4(1.6)	5(2.0)	4(1.6)	
Hyperlipidemia	With	0(0)	0(0)	1(12.5)	0(0)	0.213
	Without	24(9.5)	4(1.6)	4(1.6)	4(1.6)	
Radiotherapy	With	13(8.2)	2(1.3)	4(2.5)	3(1.9)	0.821
	Without	10(10.4)	2(2.1)	1(1.0)	1(1.0)	

EF = ejection fraction; n = number; CHF = congestive heart failure; ICU = intensive care unit

* Taxane included docetaxel and paclitaxel

**Fig 2:**
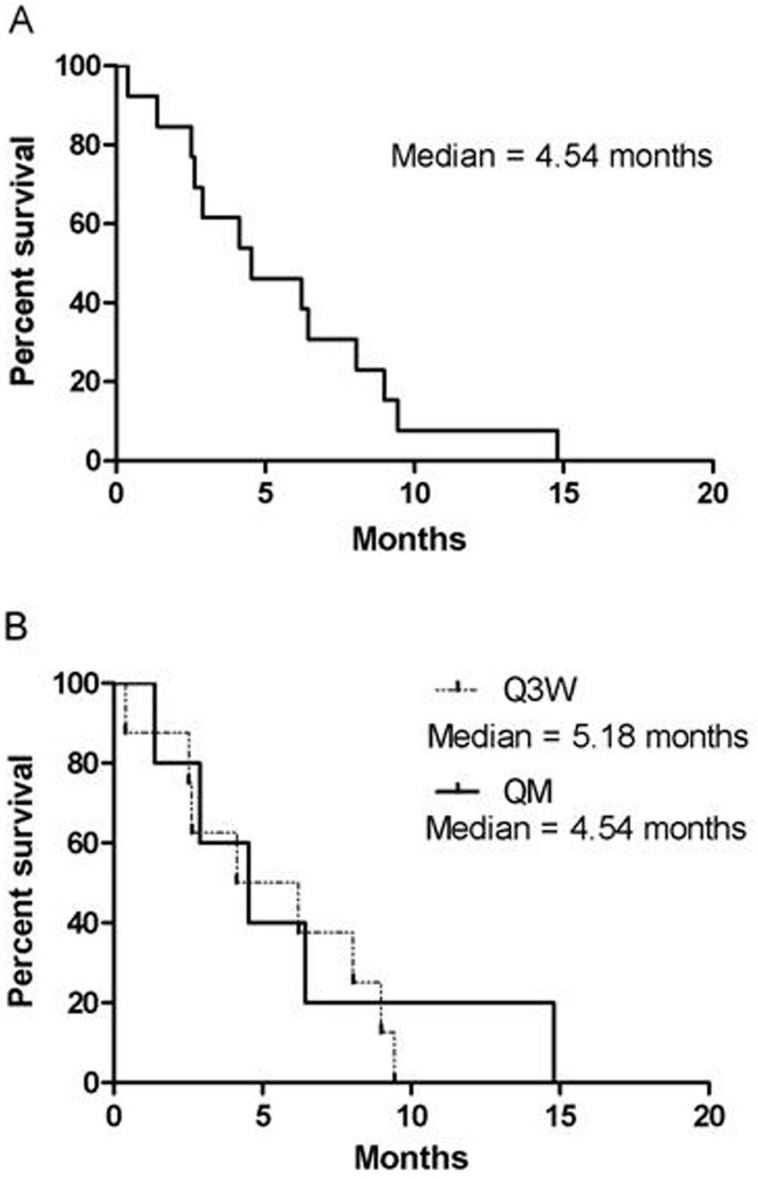
Time to cardiac events (grade ≥II). (A)The median time to cardiac events more or equal to grade II is 4.54 months, (B)There is no significant difference between the Q3W and QM groups.

The median time to significant CV dysfunction (≥grade II toxicity) was 4.54 months, as calculated using a Kaplan-Meier plot (Fig 2A); there was no difference between the Q3W and QM groups (Fig 2B, *p* = 0.71). The median duration until LVEF recovery to more than 50% was 9.5 months. However, four patients (30.8%) still suffered from symptomatic CHF despite medical aids and cessation of trastuzumab use. Angiotensin-converting-enzyme inhibitors (ACEIs), β-blockers, and diuretics were used to treat CHF (Table 3).

**Table 3:** The clinical characteristics, cardiac evaluation and treatment in patients with significant cardiac dysfunction (≥ grade II toxicity).

Case	Age (years)	Stage	Group	CT	Trastuzumab interval	CV toxicity (grade)	Time to CV event (month)	EF % change (lowest /highest)	Time to EF≥50% (month)	Trastuzumab Re-use	Medication
1	64	IIIC	Adjuvant	A→T	Q3W	3	8.98	30/62	9.0	No	ACEI, diuretics, Ubiheart
2	73	IIB	Adjuvant	CMF	QM	3	1.38	30/57	7.0	No	β-blocker, diuretics
3	63	IIIA	Adjuvant	E→T	Q3W	3	2.53	20/35	NR	No	ACEI
4	45	IIB	Adjuvant	A→T	Q3W	4	9.44	15/60	14.0	No	ACEI, β-blocker, diuretics, Ubiheart
5	32	IIIC	Adjuvant	E→T	QM	2	4.54	50/67	1.0	Yes	No
6	59	IIIA	Adjuvant	A→T	Q3W	4	2.63	40/60	7.0	No	ACEI, β-blocker, diuretics, Ubiheart
7	55	IIIA	Adjuvant	A→T	Q3W	2	4.14	20/64	9.5	No	ACEI, β-blocker, diuretics
8	51	IIB	Adjuvant	A→T	Q3W	4	0.39	20/60	20.0	No	Ubiheart
9	54	I	Adjuvant	T	Q3W	2	6.21	50/66	3.0	Yes	No
10	41	I	Adjuvant	A	QM	2	2.89	45/63	3.0	No	ACEI
11	47	IV	Metastases	A	QM	4	14.79	30/45	NR	No	β-blocker, diuretics, Ubiheart
12	55	IV	Metastases	A→T	Q3W	3	8.05	29/45	NR	No	β-blocker
13	66	IV	Metastases	A	QM	3	6.44	15/25	NR	No	ACEI, diuretics

CT: chemotherapy, CV: cardiovascular, EF: ejection fraction, A→T: doxorubicin to taxane, E→T: epirubicin to taxane, T: taxane, A: doxorubicin, CMF: cyclophosphamide/methotrexate/fluorouracil, NR: no recovery, ACEI: angiotensin-converting-enzyme inhibitor, Ubiheart: ubidecarenone

## Discussion

Several clinical trials have demonstrated that trastuzumab significantly enhances therapeutic response and reduces the risk of recurrence for patients with either metastatic or early-stage HER2-overexpressing BC [13–15]. Patients in these studies either received a loading dose of 4 mg/kg intravenously, followed by a weekly infusion of 2 mg/kg, or were placed on a triweekly protocol with a loading dose of 8 mg/kg, followed by 6 mg/kg every 3 weeks for 1 year in adjuvant therapy or a longer period in metastatic setting.

In Taiwan, one vial of trastuzumab contains 440 mg. According to the policies of our National Health Insurance Administration, some early-stage BC patients might need to pay for trastuzumab out of pocket. Therefore, we decided to simplify the infusion protocol by decreasing the frequency of infusions in our patients. In our study, we have demonstrated that a monthly fixed-dose of 440 mg trastuzumab was effective in both adjuvant and metastatic settings. In our metastatic cohort, the median time to progression was 15.35 months versus 9.93 months for the fixed-dose versus the weighted-dose protocol (*p* = 0.23; Fig 1A). Since this is a retrospective study, the trastuzumab treatment may be the first or one of several lines of anti-cancer treatment in these patients. Treatment response was good compared with previous trials, which included both first-line and multiple prior lines of treatment, ranging from 2 to 11 months [16]. The median OS was also good in both groups, 53.16 months versus 39.06 months in the QM versus Q3W groups (*p* = 0.19; Fig 1B). In the adjuvant cohort, we found that fixed-dose protocol was feasible and effective for clinical practice; the medians of both PFS and OS were not reached.

HER signaling can modulate the myocardial response to chemotherapy-induced injury and may worsen anthracycline-related cardiotoxicity [17]. The North Central Cancer Treatment Group N9831 adjuvant breast cancer trial showed that the 3-year cumulative incidence of CHF was 2.8% to 3.3% [7]. The incidence of CHF requiring medication in our cohort was 3.4%. Six of these patients were in the Q3W group, and three were in the QM group. The incidence of CHF in our trial was lower than that in some previous clinical trials (4.7%) [16]. However, very few researchers have ever analyzed the effect of dosage differences on cardiotoxicity. Belkacémi et al. [9] found that weekly infusions of trastuzumab lead to a higher rate of LVEF decrease. Combined with our data, we can postulate that increasing the trastuzumab dose while prolonging the interval between scheduled infusions will not increase the incidence of cardiac events.

Whether radiotherapy increases cardiac mortality is still controversial [18, 19]. In our metastatic and adjuvant cohorts, both radiotherapy and lesion side did not correlate with cardiac myopathy. Some have suggested that modern radiotherapy techniques markedly reduce cardiac risk, but long-term follow up is needed. Several studies have evaluated the correlation between trastuzumab-related CHF and possible CV risk factors, including age, hypertension, anthracycline-containing regimens, smoking, and diabetes [20]. In our study group, we found that patients aged less than 55 years might have a higher incidence of cardiac events (15.3% versus 12.5%, *p* = 0.032). It is possible that a higher proportion of our younger patients received anthracycline-containing therapy, which may worsen cardiac toxicity (76.2% versus 65.5%, *p* = 0.021). Russo et al. [21] found that patients with hypertension had a relatively higher incidence of trastuzumab-related CHF. They also found that onset of CHF predominantly occurred in the first 6 months of the treatment, which was similar to the findings from our group (median onset of cardiac events 4.54 months; Fig 2A). Therefore, we suggest that for higher-risk groups we should monitor cardiac function much more intensively during the first year of trastuzumab administration.

In our study, the trastuzumab discontinuation rate was 4.2%, compatible with the results of previous clinical studies (4.2% in the HERA trial to 17.0% in the N9831 trial). Most of our patients were treated with diuretics, ACEIs, β-blockers, and other drugs. Nine of 13 patients (69.2%) responded to these therapies with obvious symptomatic improvement and LVEF recovery to more than 50%. Two patients with initial asymptomatic grade II cardiotoxicity received a trastuzumab rechallenge and did not experience any episodes of heart failure or toxicity of more than grade I severity. In current clinical practice, trastuzumab rechallenge is generally recommended primarily in the metastatic setting or following careful evaluation of risks and benefits after LVEF returns to normal [20]. Ubidecarenone, also known as coenzyme Q_10_ (CoQ_10_), is a powerful antioxidant, and reduced concentrations of myocardial tissue CoQ_10_ is associated with the severity of CHF. Mortensen et al. reported that concurrently administering CoQ_10_ and standard therapy for CHF patients could improve symptoms and reduce CV mortality, but it could not significantly improve LVEF [22]. There were five patients who received CoQ_10_ treatment in our analysis. Four had severe grade IV events and one experienced grade III heart failure. However, only one patient, case #11, received long-term CoQ_10_ treatment due to the uncontrolled CHF. The rest of the patients stopped taking CoQ_10_ when LVEF recovered or the CHF was well controlled. Currently, there is limited data about the role of CoQ_10_ in the treatment of trastuzumab-induced cardiotoxicity. More prospective studies are necessary.

Currently, increasing health care costs must be considered, and cost-effectiveness analyses are used frequently to assist government payers to make decisions about whether the therapy is worthwhile. Trastuzumab is a relatively expensive drug. Several cost-effectiveness analyses support the use of trastuzumab in both early and metastatic BC patients [23, 24]. Ward et al. [25] suggested that the dosing protocol used in the FinHer study [14], with its shortened regimen, might be equally effective and be more convenient for patients. Although there were no head-to-head comparisons of the 9 week and 12 month treatments, the protocol used in the FinHer study proved to be effective [26]. In Taiwan, we only have one dosage of trastuzumab available, with 440 mg per vial. The 150 mg vials that are available elsewhere are not sold here. In the Q3W group, averagely 18.2% of trastuzumab dispensed for each cycle in the adjuvant setting was discarded, as well as 23.8% was wasted in the metastatic setting according to our analysis. Therefore, using a fixed-dose of 440 mg of trastuzumab every month is not only very convenient to patients and pharmacists, but it can also reduce waste of a costly drug. In the pharmacoeconomic point of view, for example, a 60-kilogram woman receives adjuvant trastuzumab therapy with 360 mg dosage every three weeks for totally 18 cycles. The cost is around NT$ 60,000 for each cycle, including the waste. Using 440 mg fixed dosage every month, the cost for each cycle is the same, but the total expense will be 33% off because only 12 cycles are needed. In patients with metastatic breast cancer, the median treatment courses in our cohort for both groups are 15 cycles, therefore the cost is the same, but the treatment period is longer in QM group.

There are several limitations of our study owing to its retrospective design, including heterogeneity of patients and disease status, lack of pharmacokinetic information, different chemotherapeutic protocols and existence of selection bias yielded by patients and physicians. It raises a concern that these two dosing schedules cannot be compared equally and properly. Consequently, we only demonstrate the feasibility and effectiveness of the fixed-dose monthly protocol in this study. Further large randomized prospective are warranted to confirm the relative merits of these two dosing schedules. The initial trastuzumab pharmacokinetic studies suggested that the half-life was 6–8 days. However, plasma levels with tri-weekly dosage are similar to those with conventional weekly dosage, which can prove that chronic administration prolongs the elimination half-life of trastuzumab [27]. Therefore, we believe that a fixed-dose monthly schedule should be feasible to maintain the effective serum trastuzumab level. Besides, biomarkers were not evaluated in our study. Some studies reported that elevated high-sensitivity C-reactive protein, cardiac troponin I, and troponin T appeared to predict trastuzumab-induced cardiotoxicity [20, 28, 29]. However, regular follow-up of the biomarkers is not suggested because conflicting results of these biomarkers existed among the previous trials.

In conclusion, fixed-dose regimens of trastuzumab are simple and feasible for daily clinical practice. CV risk is not higher than in previous clinical trials. The regimen is also economically feasible. Larger-scale studies with longer-term follow up are needed to confirm our results.
